# Role of EGFR expressed on the granulosa cells in the pathogenesis of polycystic ovarian syndrome

**DOI:** 10.3389/fendo.2022.971564

**Published:** 2022-11-11

**Authors:** Jun-Hui Zhang, Lei Zhan, Ming-Ye Zhao, Jin-Juan Wang, Fen-Fen Xie, Zu-Ying Xu, Qian Xu, Yun-Xia Cao, Qi-Wei Liu

**Affiliations:** ^1^ Department of Obstetrics and Gynecology, The First Affiliated Hospital of Anhui Medical University, Hefei, Anhui, China; ^2^ National Health Commission (NHC) Key Laboratory of Study on Abnormal Gametes and Reproductive Tract, Anhui Medical University, Hefei, Anhui, China; ^3^ Key Laboratory of Population Health Across Life Cycle, Anhui Medical University, Ministry of Education of the People’s Republic of China, Hefei, Anhui, China; ^4^ Department of Obstetrics and Gynecology, The Second Affiliated Hospital of Anhui Medical University, Hefei, Anhui, China; ^5^ Interventional Operating Room, Weihai Central Hospital, Weihai, Shandong, China; ^6^ Department of Gynecological Minimal Invasive Center, Beijing Obstetrics and Gynecology Hospital, Capital Medical University, Beijing, China; ^7^ Beijing Maternal and Child Health Care Hospital, Beijing, China; ^8^ Department of Histology and Embryology, Anhui Medical University, Hefei, Anhui, China

**Keywords:** polycystic ovarian syndrome (PCOS), granulosa cells, epidermal growth factor receptor, dehydroepiandrosterone (DHEA), pathogenesis, ovarian function

## Abstract

Polycystic ovarian syndrome (PCOS) is one of the most common endocrinological disorders affecting between 6 to 20% of reproductive aged women. However, the etiology of PCOS is still unclear. Epidermal growth factor receptor (EGFR) plays a critical role in the growth and development of ovarian follicles. In our previous study, we showed that the expression level of EGFR was significantly higher in the cumulus granulosa cells from women with PCOS than that of normal women, suggesting that EGFR may play a potential role in the pathogenesis of PCOS. The present study further evaluated the association between EGFR and PCOS through both in clinical observation and animal experiments. We firstly validated the differential expression of EGFR in cumulus granulosa cells between PCOS patients and normal subjects by qRT-PCR and immunofluorescence staining. Then we generated a mouse model (n=20) of PCOS by injecting dehydroepiandrosterone (DHEA). The PCOS mice were then injected with an E corpus GFR inhibitor (AG1478) (n=10), which significantly improved the sex hormone levels in the estrous cycle stage, and the serum levels of LH, FSH and testosterone were compared with the PCOS mice without EGFR inhibitor treatment (n=10). Decreasing the expression level of EGFR in the PCOS mice also improved the ovulatory function of their ovaries which was indicated by the multifarious follicle stage in these mice as compared with the PCOS mice without EGFR inhibitor treatment. Also, the number of corpopa lutea were higher in the control group and the EGFR inhibitor treated group than in the PCOS group. The sex hormone levels and reproductive function were not significantly different between the control mice and the PCOS mice treated with the EGFR inhibitor. Our results demonstrated that EGF/EGFR signaling affected the proliferation of cumulus granulosa cells, oocyte maturation and meiosis, and played a potential role in the pathogenesis of PCOS. Therefore, the selective inhibition of EGFR may serve as a novel strategy for the clinical management of PCOS.

## Introduction

Polycystic ovarian syndrome (PCOS) is the one of the most common neuro-endocrine dysfunctions of the hypothalamic-pituitary-ovarian (HPO) axis and metabolic disorders in reproductive women, and is characterized by hyperandrogenism, ovulatory dysfunction and polycystic ovarian morphology (1). Studies have shown that the HPO axis plays a vital role in endocrine dysfunction of PCOS such as abnormal GnRH pulse frequency, increased luteinizing hormone (LH)/follicle-stimulating hormone (FSH) ratio, and adrenal and ovarian androgen excess (2). Increased GnRH pulsatility triggers elevated synthesis and secretion of LH from the pituitary, leading to elevated LH/FSH ratios, which is the hallmark of PCOS (3). PCOS affects between 6% to 20% of women in their reproductive age, causing infertility, amenorrhea, obesity, chronic inflammation, metabolic syndrome, cerebrovascular events and so on (4, 5). The etiology of PCOS is complex and still unclear. Genetic susceptibility, androgen exposure in early life, adiposity related dysfunction, environmental factors, vitamin D deficiency and insulin resistance may play a causal role in the pathogenesis of PCOS (6, 7). The increased density of small antral follicles (5-8 mm) in women with PCOS are growth arrested and cannot undergo further maturation or participate in ovulation as compared with normal women, which are the main characteristic features of PCOS (5). The excess androgen in women with PCOS has been demonstrated to affect the development of primordial follicles into antral follicles. However, androgen excess also hinders ovulation by impairing the selection of dominant follicles (8–10).

Epidermal growth factor receptor (EGFR) located on granulosa cells is activated by EGF-like peptides and plays an important paracrine role in the growth and development of the vertebrate ovarian follicle (11). EGF-like peptides are triggered when LH released by the anterior pituitary binds to its receptor, and this signaling cascade is regulated by the HPO axis, leading to the final phase of oocyte differentiation (12). LH released by the anterior pituitary binds to the LH receptor on the mural granulosa cells, triggering the release of EGF-like peptides and activating EGFR expressed on the granulosa cells. Activation of the EGF/EGFR signaling cascade increases the expression of MAPK, which promotes the phosphorylation of connexin 43 (GJA1), causing the closure of gap junctions (11). On the other hand, activated EGFR also inhibits the production of cyclic guanosine monophosphate (cGMP) and suppresses meiosis (13). This is because cGMP triggers meiosis in oocytes by inhibiting the hydrolysis of cyclic adenosine monophosphate (cAMP). Therefore, hyper-stimulation of the EGFR axis inhibits meiosis of the oocytes (13). Importantly, several studies have suggested that EGFR is associated with the expansion of granulosa cells, and in the induction of meiotic maturation of the oocyte and ovulation. One previous study found that the blockage of EGFR induced apoptosis in granulosa cells (14). A recent study reported that folliculogenesis was blocked at the primary-secondary growth transition in zebrafish mutants upon EGFR knockout by CRISPR/Cas9 technique, leading to female infertility (15). In cattle ovary, studies identified that the protein expression of EGFR was higher in the antral follicles than that in other follicular growth stages, which indicates that EGFR may promote follicular development during follicles growth waves (15).

In our previous study, we showed that the expression level of EGFR was significantly higher in the cumulus granulosa cells from women with PCOS women than from normal women (16). Since EGF/EGFR dominated the process of follicular growth and development, which was inhibited in PCOS, we speculated that EGFR played a potential role in the pathogenesis of PCOS. Our present study aims to further examine the association between EGFR and PCOS, wherein we hypothesized that the abnormal expression of EGFR may cause the occurrence of PCOS. Here, we firstly validated the differential expression level of EGFR in granulosa cells between PCOS patients and patients without PCOS by qRT-PCR and immunofluorescence. These results verified there was a significant difference in the expression of EGFR in granulosa cells from PCOS patients and normal subjects. Then we further developed a mouse model of PCOS and used it to investigate the role of EGFR in the development of PCOS. Our results provide new insights into the association of EGFR with the incidence of PCOS.

## Materials and methods

### Study population and selection of granulosa cells

8 women with PCOS and 8 women without PCOS were included in this study. We collected granulosa cells from 16 patients who were willing to donate several granulosa cells from the Cumulus cell-oocyte complexes (COCs) and undergoing treatment with assisted reproductive technology (ART) at The First Affiliated Hospital of Anhui Medical University. The study protocol was approved by the Research Ethics Committee of Anhui Medical University (No. 20170046) and was conducted in accordance with the institutional guidelines. All the participants gave written informed consent. Clinical characteristics of patients such as age, body mass index (BMI), LH, FSH and androstenedione were recorded. PCOS was diagnosed based on the Rotterdam 2003 criteria: hyperandrogenism, oligoovulation and/or anovulation, and polycystic ovaries. We excluded patients with Cushing’s syndrome, congenital adrenal hyperplasia, and androgen-secreting tumors. Control patients received ART treatment for tubal disease but they had regular menstrual cycles, normal levels of hormone, and normal ovarian morphology. COCs were isolated *via* ultrasound-guided vaginal puncture and washed in phosphate-buffered saline (PBS). Granulosa cells were selected from the COCs, and prepared for further analysis by reverse-transcriptase polymerase chain reaction (qRT-PCR) and immunofluorescence.

### RNA extraction and analysis by quantitative real time polymerase chain reaction

RNA was extracted from granulosa cells and converted into cDNA according to the Smart-seq2 protocol (17). cDNA was amplified and purified twice by using AMPure XP beads and 80% ethyl alcohol after the first strand reaction. The significantly differentially expressed genes were validated by qRT-PCR (208054, QIAGEN, Germany). Following were the sequences of the two primers: EGFR primers (sense primer, AGGCACGAGTAACAAGCTCAC; antisense primer, ATGAGGACATAACCAGCCACC) and glyceraldehyde-3-phosphate dehydrogenase (GAPDH) primers (sense primer, ACCCGCCCTATCTCAACTACC, antisense primer, AGGACACCATAATGACAGCC). qRT-PCR was performed in a total reaction volume of 25 μL, including 1 μL cDNA (1 ng/μL), 10 μL 2x SYBR green PCR master mix, 0.1 μL QN ROX reference dye, 1 μL forward primer (10 mmol/L), 1 μL reverse primer (10 mmol/L), and 6.9 μL RNase-free water. The PCR initial activation was achieved by heating the samples to 95°C for 2 min, followed by a total of 40 cycles of denaturation at 95°C (5 s) and 60°C (30 s).

### Immunofluorescence

Granulosa cells isolated from the participants were washed thrice with PBS and then fixed for 10 min at room temperature in 4% paraformaldehyde. After 10 min, the granulosa cells were washed thrice with 0.5% Triton-100 and incubated with 0.5% Triton X-100 at room temperature for 40 min. Then, the cells were washed thrice with PBS and then blocked with 3% normal goat serum for 60 min. All reagents were made into 50ul droplets and transferred of granulosa cells with capillary tubes. Following this, the cells were washed once and then incubated with the primary antibody (1: 50 dilution, ab32077, abcam) at 4°C overnight. Then, the cells were washed thrice with PBS and incubated with antrabbitimmunoglobulin G fluorescein secondary antibody (1:100 dilution, 4414s, Cell Signaling) for 1 h at room temperature. Afterwards, the cells were washed thrice with PBS and the DNA in the cells was labeled with DAPI for 10 min. Finally, the granulosa cells were placed in a drop of antifade mounting medium for fluorescence and observed under a fluorescence microscope.

### Animals and establishment of the mouse model of PCOS

Thirty female C57BL/6J mice (21 days old) were purchased from SPF (Beijing) Biotechnology Co., Ltd. The mice were maintained in a pathogen-free facility with a 12-hour light-dark cycle, according to the institutional guidelines of the Animal Care and Use Committee. All animal studies were approved by the Anhui Medical University (No. LLSC20170062). We performed a 5-day vaginal smear on the purchased animals to ensure that the mice used for the experiment had a regular sexual cycle. We picked out mice from proestrus stages as our experimental samples to ensure that initially all the mice were in the same estrous cycle. Then these mice were randomly divided into 3 groups (n=10 each group): the control group, DHEA group, DHEA+EGFR inhibitor group. The PCOS mouse model were subjected to daily subcutaneous injection of DHEA (Solarbio, 6mg/100g body weight, dissolved in 0.09 ml sesame oil and 0.01 ml 95% ethyl alcohol) for 20 days and intraperitoneal injected with normal saline (100 uL/20g body weight) for 7 days. The control group were injected with sesame oil for 20 days and intraperitoneal injected with normal saline for 7 days at the same time. Then the DHEA+EGFR inhibitor group were subjected to daily subcutaneous injection of DHEA (Solarbio, 6mg/100g body weight, dissolved in 0.09 ml sesame oil and 0.01 ml 95% ethyl alcohol) for 20 days and intraperitoneal injected with EFGR-inhibitor (AG1478) for 7 days (10 mg/kg/day) (18, 19).

### Identification of the estrous cycle stage

On approximately the 20th day after the first treatment to establish the PCOS mice model, the estrous cycle of the mice was evaluated by vaginal cytology for 10 consecutive days. Mouse secretions were obtained from the vagina by using a cotton swab dipped in PBS, then spread evenly on the slide. Meanwhile cell morphology and estrous cycles were observed by phase-contrast microscopy (Nikon, Japan).

### Histological analysis

At the end of the experiment, we obtained ovaries of mice at proestrus stages according to the results of vaginal smear ovarian tissues of the 3 groups mice (n=10 each group) were fixed in 4% paraformaldehyde overnight and then embedded in paraffin. Sections of 5 μm thickness were stained with hematoxylin and eosin. The images were captured by a inverted fluorescence microscope, bar=200μm (Nikon,TE2000U, Japan).

### Serum analyses

The levels of sex hormones were tested at proestrus stages. The blood samples were collected from 3 group mice (n=10 each group) by cardiac puncture after mice were anesthetized. The blood was put into 1.5 ml tubes for 2 hours in room temperature to clot and centrifuged at 2000 rpm for 15 min. The serum was collected in the -80 refrigerator. The levels of serum gonadal hormones including LH (Luteinizing Hormone), T(testosterone) and FSH (Follicle-Stimulating Hormone) were determined using commercial ELISA kits according to the directions (n=5 each group). The detection limit was 0.31 ng/mL for T, 1.56 ng/ml for FSH, 0.31 ng/ml for LH. The variation was performed using three samples, low concentration samples, medium concentration samples and high concentration samples. The intra-assays were performed 20 times on 1 plate, respectively. The inter-assays were performed 20 times on 3 plates, respectively. The intra-assay variation was 4.27% to 8.13% and the inter-assay variation was 6.18% to 9.23% for T. The intra-assay variation was 4.49% to 6.38% and the inter-assay variation was 4.23% to 5.88% for FSH. The intra-assay variation was 4.64% to 4.94% and the inter-assay variation was 4.64% to 5.62% for LH. [ELABSCIENCE (E-EL-0155c, E-EL-M0511c, and E-EL-M3053, respectively)].

### Ovarian morphology

To assess changes in ovarian tissue, structures within the ovary were morphologically divided into cystic follicles and corpora lutea. Follicles were classified as cystic follicles if they possessed a single large fluid-filled cyst. Corpus luteum was constituted of residual follicular wall cells containing granulosa and theca cells after ovulation. As expected, DHEA caused ovarian dysfunction, as judged by a decreased number of corpora lutea and an increased number of cystic follicles. We selected the largest section of ovarian tissue and every third section was counted for each mouse, and we selected 3 neighboring slices containing the corresponding equatorial section and the mean values of cystic follicles and corpora lutea in the three sections were considered to represent the ovarian function of each mouse.

### Statistical analysis

Descriptive statistics were used to describe characteristics of the three groups. All statistical analyses were performed using the SPSS 22.0 (IBM SPSS Statistics for Macintosh, Version 22.0 Armonk, NY : IBM Corp.). Graphs were generated by using GraphPad Prism 9 software. Data were presented as mean ± SEM. The data among the 3 groups were analyzed using an one-way ANOVA, followed by Fisher’s least significant difference (LSD) method for multiple comparisons. *P*<0.05 indicated statistical significance.

## Results

### Clinical characteristics of PCOS and control patients

There was significant differences between PCOS patients and control patients in LH, LH/FSH ratio and testosterone, while FSH, age and BMI were no difference between these two groups (**Table 1**). Patients with PCOS had increased LH (13.4 ± 1.3 uIU/ml) compared with control patients (5.9 ± 0.8 uIU/ml) (*P*<0.001). Patients with PCOS had a higher LH/FSH ratio and testosterone than control patients LH/FSH ratio (6.8 ± 0.6 vs 3.5 ± 0.5, *P*<0.001) and testosterone (1.9 ± 0.2 nmol//L vs 0.9 ± 0.1 nmol//L, *P*<0.001).

**Table 1:** Clinical characteristics and outcome of patients with and without PCOS.

Variables	Non-PCOS (*n* = 8)	PCOS (*n* = 8)	*P* value
Age (year)	26.4 ± 1.1	25.9 ± 0.8	0.7035
BMI (kg/m2)	23.6 ± 0.1	23.1 ± 1.7	0.7804
AFC	7.8 ± 0.5	>24.000	<0.001
FSH (IU/L)	7.4 ± 0.7	7.6 ± 0.3	0.7820
LH (IU/L)	5.9 ± 0.8	13.4 ± 1.3	<0.001
LH/FSH	3.5 ± 0.5	6.8 ± 0.6	<0.001
Insulin (IU)	12.4 ± 1.7	15.4 ± 4.4	0.8541
Testosterone (nmol/mL)	0.9 ± 0.1	1.9 ± 0.2	<0.001
Androstenedione (nmol/mL)	1.8 ± 0.1	2.3 ± 0.3	0.1611
SHBG (nmol/mL)	54.2 ± 7.0	40.0 ± 4.8	0.1177

BMI, body mass index; FSH, follicle-stimulating hormone; LH, luteinizing hormone.

AFC, antral follicle; SHBG, sex hormone-binding.

### EGFR expression in human cumulus granulosa cells

**Figure 1:**
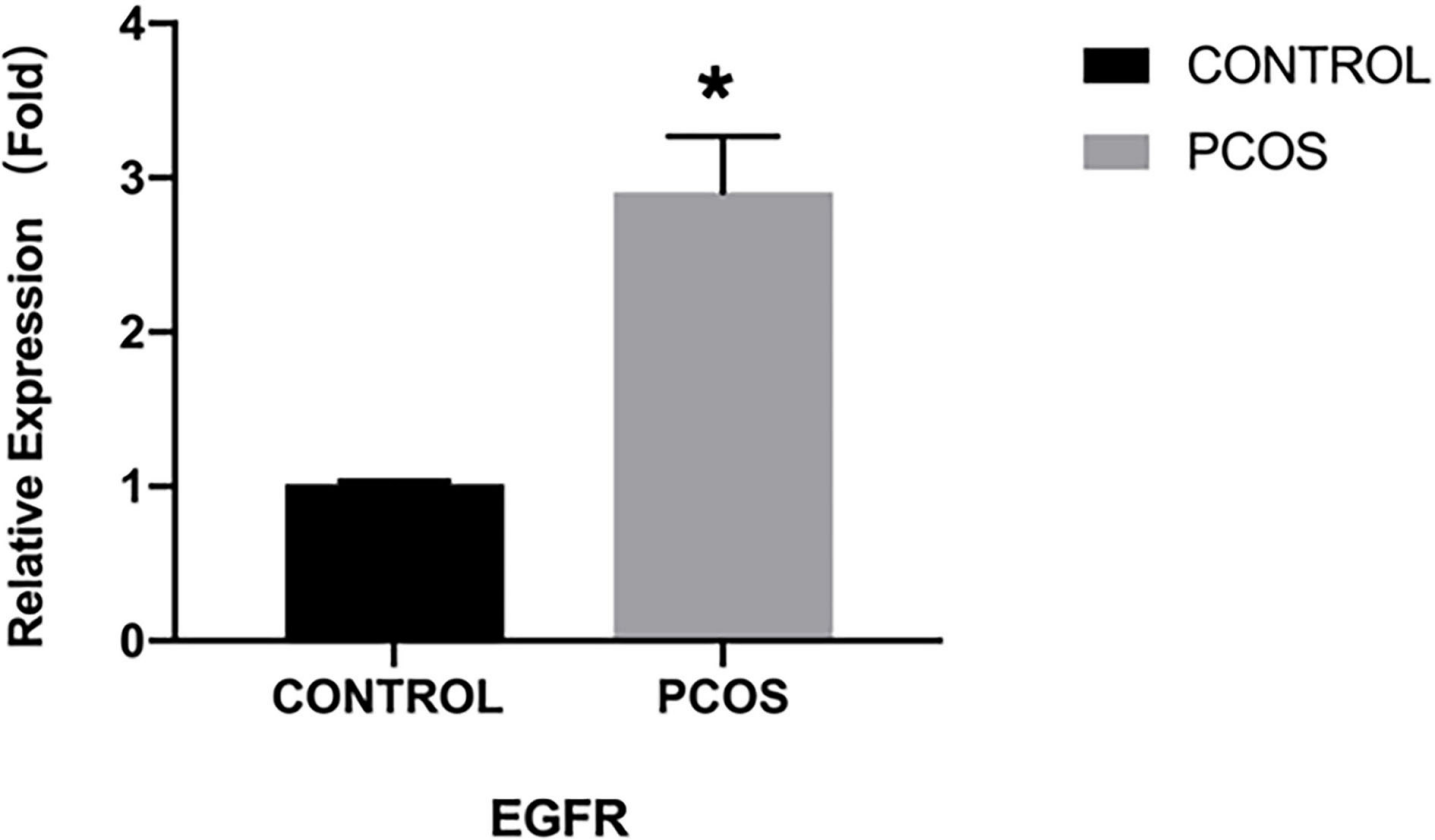
EGFR expression demonstrated by qRT-PCR in PCOS women (n=8) and normal women (n=8). *P < 0.05 vs CONTROL.

**Figure 2:**
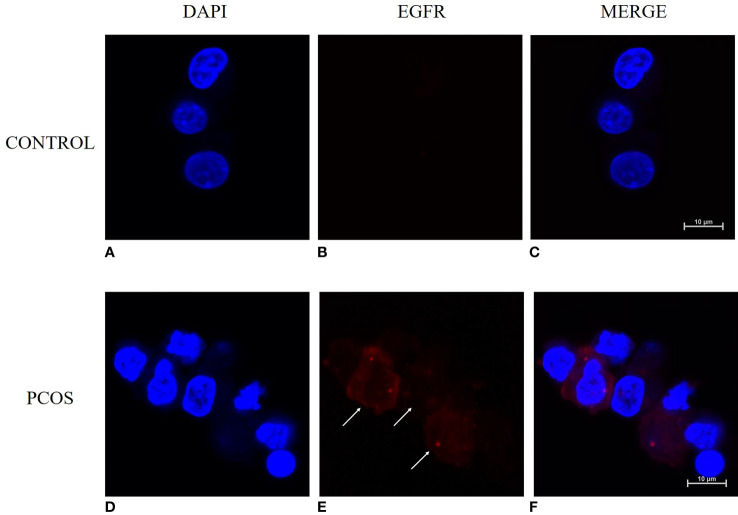
Immunofluorescence staining for detecting the expression pattern of EGFR in human granulosa cells with and without PCOS (n=8). **(A–C)** granulosa cells from women without PCOS. **(D–F)** granulosa cells from PCOS women. Granulosa cells were counterstained with EGFR (red) and nuclei (DAPI, blue). Bar = 10μm.

The results from qRT-PCR analysis of granulosa cells from women showed a significantly higher expressing of EGFR in granulosa cells from the PCOS group than the control group (*P*<0.05) (**Figure 1**). Immunofluorescence analysis was also performed on granulosa cells from women to detect the expression pattern of EGFR. While we detected a positive fluorescence signal for EGFR in samples from the PCOS group, the fluorescence signal from EGFR was much weaker in the control group (**Figure 2**).

### Inhibiting EGFR expression leads to improvement in the levels of sex hormones in PCOS mice

**Figure 3:**
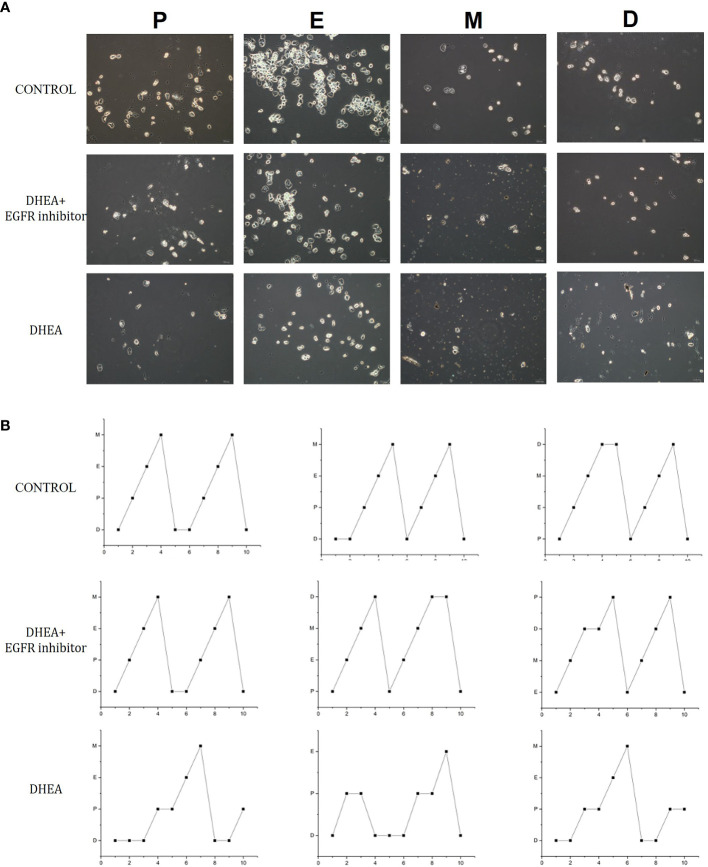
The effect of EGFR-inhibitor on estrous cycles in DHEA-induced PCOS mice. **(A)** 10 consecutive days observation in each group (n=10) for vaginal cytology unstained by phase contrast microscope. **(B)** The effect of EGFR-inhibitor on estrous cycle in DHEA-induced PCOS mice. P, proestrus; E, estrum; M, metestrus; D, anoestrum.

**Figure 4:**
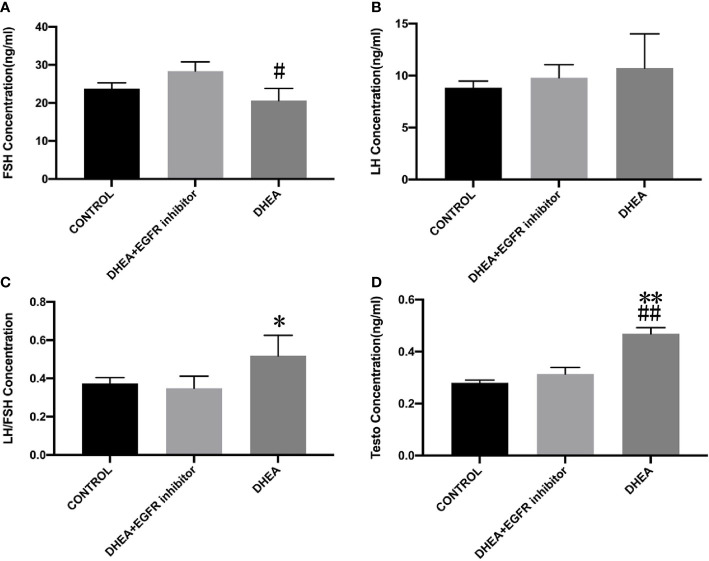
The effect of EGFR-inhibitor on levels of serum sex hormones in DHEA-induced PCOS mice (n=10). Data are presented as mean ± SEM. **(A)** The serum level of FSH (^#^P=0.017 vs DHEA+EGFR inhibitor) **(B)** The serum level of LH (P>0.05). **(C)** LH/FSH (*P=0.05 vs CONTROL). **(D)** The serum level of testosterone (**P=0.002 vs CONTROL; ^##^P=0.003 vs DHEA+EGFR inhibitor).

Smear tests showed that DHEA group mice had an acyclic oestrus cycle pattern compared with control group, whereas DHEA+EGFR inhibitor group mice had regular oestrus cycle pattern especially the typical estrus period produced. Additionally, in DHEA+EGFR inhibitor group, typical cornified epithelial cells appeared, indicating that the mice recovered estrus after treatment (**Figure 3**). As shown in **Figure 4**, the serum levels of LH, FSH and testosterone were tested in mice from all the three groups. There was a significant difference between three groups in terms of FSH and testosterone concentrations in the serum. Mice in the control group and the DHEA+EGFR inhibitor treated group had significantly higher FSH concentrations than in the DHEA induced group (*P*<0.05) (**Figure 4**). We found that there was no significant difference of LH concentration between the control group and the EGFR inhibitor treated group (**Figure 4**), while the LH/FSH ratio was higher in the DHEA induced group than in the control group and the DHEA+EGFR inhibitor treated group (**Figure 4**). The serum levels of testosterone were significantly elevated in the DHEA induced group as compared with the control group. However, when PCOS mice were treated with EGFR inhibitor, testosterone concentrations were restored to normal levels, which was comparable to the normal group (**Figure 4**).

### Inhibiting EGFR expression promotes ovarian function in PCOS mice

**Figure 5:**
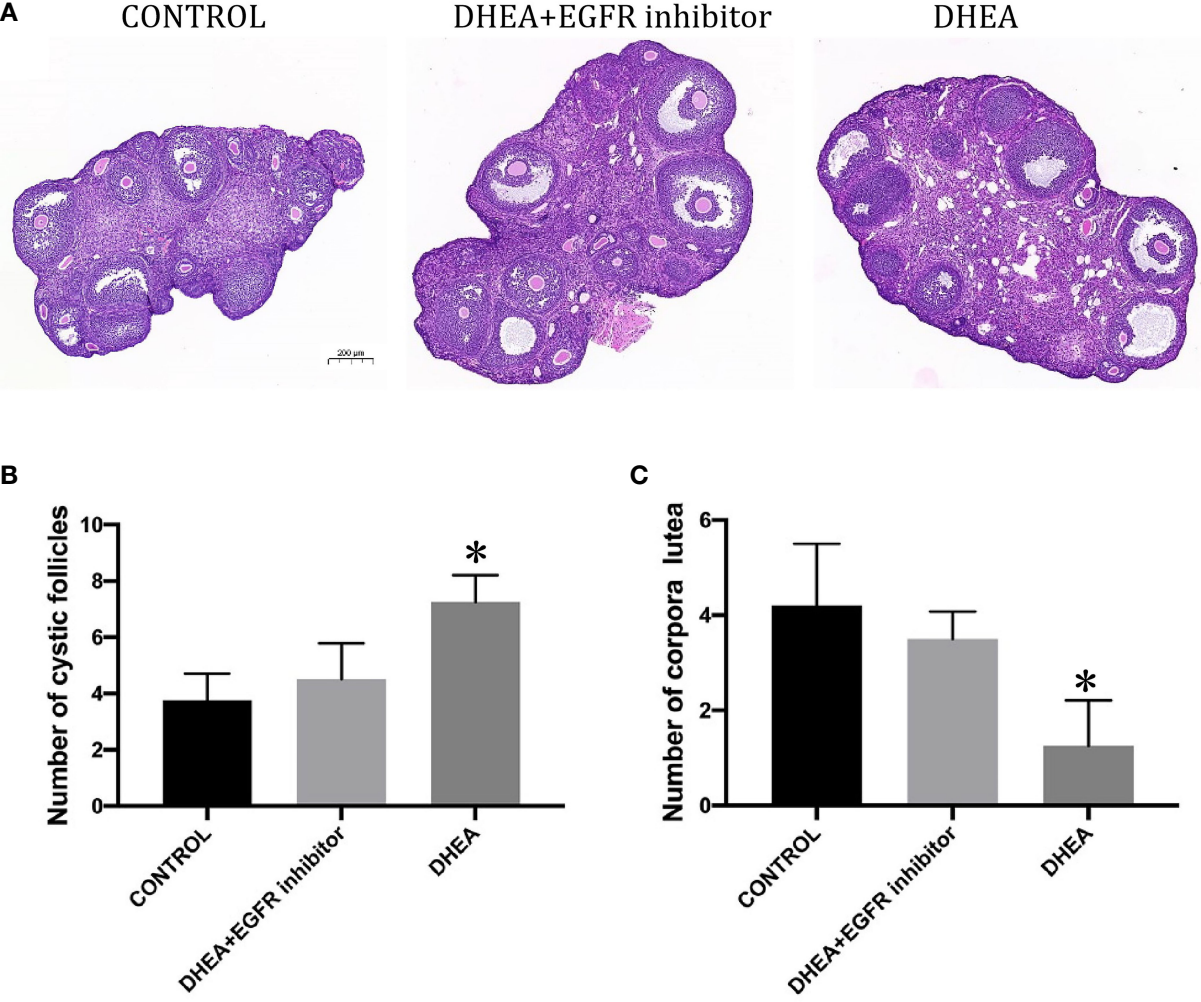
The effect of EGFR-inhibitor on ovarian morphology and total number of cystic follicles and CLs per half ovary in DHEA-induced PCOS mice (n=10). **(A)** HE staining of ovary (Bar=200ìm). **(B)** Number of cystic follicles (*P<0.05 vs CONTROL). **(C)** Number of corpora lutea (*P<0.05 vs CONTROL).

Histopathological images of ovarian sections were taken with an optical microscope, and microscopic views of the ovarian sections are shown in **Figure 5**. The area of the ovaries was significantly different between the study groups. A one-way between-groups analysis showed that there was a significant difference between DHEA group and control group, but not for DHEA+EGFR inhibitor group. As shown in **Figure 5**, the number of cystic follicles in the DHEA group were significantly higher than the control group and the DHEA+EGFR inhibitor group (*P*<0.05), but there was no difference in the number of cystic follicles between the control group and the DHEA+EGFR inhibitor group (**Figure 5**), indicating EGFR inhibitor had some protective effects on ovarian function. The corpus luteum count was also significantly different between the study groups. The number of corpora lutea were higher in the control group and the DHEA+EGFR inhibitor group than in the DHEA group (*P*<0.05) (**Figure 5**).

## Discussion

Polycystic ovary syndrome (PCOS) is a common endocrine disorder in women, with a prevalence of 17.8% in reproductive age women. PCOS patients exhibit systemic complications such as infertility, metabolic disorders such as obesity, glucose metabolism disorders, and psychological disorders such as depression, which seriously endanger women’s health (20). Among them, infertility is the biggest problem that plagues women with PCOS during their reproductive years (21). PCOS is the most common cause of anovulatory infertility in reproductive age women, and about 80% of PCOS patients face infertility (21). In addition, due to the low quality of follicles, PCOS patients are also more prone to early miscarriage, fetal growth restriction and other complications during pregnancy, which seriously endanger women’s health and the health of the fetus (22). However, the pathogenesis of PCOS is complex and not well defined yet. Therefore, an in-depth study of the mechanism of follicular development abnormalities in PCOS patients is beneficial for the fundamental treatment and long-term disease management of PCOS patients, which has important scientific significance and clinical value for solving the problem of infertility in reproductive age women.

EGFR is a transmembrane glycoprotein that plays a significant role in several cellular processes such as cellular proliferation, survival, differentiation, migration and inflammation (23). Several tumors are known to exhibit increased EGFR activity, such as glioblastomas, lung cancer, breast, ovarian prostatic, pancreatic cancers, etc. EGFR is also associated with worse prognosis in some cancers such as cancer of the ovary, cervix and bladder (24). Hence, targeting EGFR may be an effective approach to treat cancer (25). EGFR may be an effective target in several clinical indications. In particular, the importance of EGFR in meiotic maturation of the oocyte has been reported in recent years. For example, Gao et al. showed that the level of serum EFG in the abortion PCOS patients was higher than that in the successful delivery PCOS patients, moreover, EFG was an independent factor which affecting and positively correlated with the pregnancy outcome of PCOS patients (26). Mechanistically, in a recent study by Wu and colleagues, they found that high expression of microRNA-194 in granulosa cell of PCOS patients could induce KGN cells apoptosis by direct targeting heparin-binding EGF-like growth factor (27). More recently, Huang and co-workers indicated that heparin-binding-EGF might induce mitochondrial dysfunction and granulosa cells apoptosis through advancing estrogen hypersecretion dependent on cAMP-PKA-JNK/ERK-Ca^2+^-FOXO1 pathway (28). Additionally, by a discovery-validation GWAS on PCOS patients and controls identifified from electronic health record using an algorithm based on Rotterdam criteria, Zhang et al. confirmed that the *ERBB4-YAP1-WWTR1* network played a potential role of EGFR and Hippo signaling in the pathogenesis of PCOS (29). Nevertheless, the relationship between EGFR and PCOS has not yet been fully investigated (15). Bidirectional communication between somatic granulosa cells and the oocyte is an essential factor for folliculogenesis, which is orchestrated by endocrine and paracrine mechanisms (30). Our study showed that the mRNA and protein expression of EGFR was higher in cumulus cells of women with PCOS than in controls. Therefore, we speculate that EGFR may be involved in promoting high expression of LH and facilitating the communication between granulosa cells and oocytes.

Then through animal experiments, we found that suppressing EGFR levels by treating PCOS mice with EGFR inhibitor promoted a huge improvement in the ovulatory function and sex hormone levels, including the oestrus cycle, LH/FSH ratio, testosterone levels, morphology of the ovary and probability of ovulation. LH released by the anterior pituitary binds to its receptor LHCGR, expressed on the surface of granulosa cells at the time of ovulation, and stimulates the release of EGF-like peptides from the ovarian somatic cells (31). LHCGR is expressed on the granulosa cells while the oocyte and the cumulus granulosa cells are not directly responsive to LH, thus, the EGF/EGFR pathway plays a vital role in mediating the communication between LH and the oocyte (32). Granulosa cells provide the physical support for developing oocyte and actively differentiated with distinct populations during folliculogenesis (33). Granulosa cells can be divided into two functionally distinct lineages: mural granulosa cells and cumulus granulosa cells (34). Cumulus granulosa cells, which are nearest around oocyte, are responsible for protecting oocyte from the microenvironment and supporting oocyte growth and maturation during follicular development through bi-directional communications between oocyte and cumulus granulosa cells (35). Therefore, our findings supported the hypothesis that EGFR played a key positive role in promoting the pathogenesis of PCOS, and that the selective inhibition of EGFR may serve as a novel strategy for the clinical management of PCOS.

Collectively, accumulating studies indicated the importance of EGFR in female reproductive system recently (36, 37). The results from our study further suggested that blocking of EGFR could prevent the progression of PCOS by recovering reproductive capacity, including inhibiting the concentrations of serum LH and testosterone, and promoting oestrus irregularities.

## Conclusion

The findings from our study indicated that EGF/EGFR signaling affected the proliferation of cumulus granulosa cells, oocyte maturation and meiosis, and might be involved in the pathogenesis of PCOS. The results from the current study suggest that the selective inhibition of EGFR could serve as a novel strategy for the treatment of PCOS in the future.

## Data availability statement

The datasets presented in this study can be found in online repositories. The names of the repository/repositories and accession number(s) can be found in the article/**Supplementary Material**.

## Ethics statement

The studies involving human participants were reviewed and approved by Research Ethics Committee of Anhui Medical University (No. 20170046). The patients/participants provided their written informed consent to participate in this study.The animal study was reviewed and approved by Anhui Medical University (No. LLSC20170062).

## Author contributions

J-HZ contributions include performing experiments, data collection and preparing figures. Q-WL was involved in study design, data analysis and writing and editing the manuscript and figures. LZ contributions include performing experiments and cells collection. M-YZ and J-JW contribution includes data collection and analysis. QX, F-FX and Z-YX contributions includes preparing figures. Y-XC contributions include study design, manuscript reviewing and editing. All authors contributed to the article and approved the submitted version.

## Funding

This work was supported by a grant from Beijing Natural Science Foundation (7214228), a fund from Beijing Obstetrics and Gynecology Hospital, Capital Medical University (FCYY201813), the National Natural Science Foundation of China (82101716), National Innovation and Natural Science Research Project of Universities in Anhui Province (KJ2020A0198), Doctoral Research Fund of Anhui Medical University (XJ202002), and Open Project of Anhui Province Key Laboratory of Reproductive Health and Genetics (RHG-2020-8).

## Acknowledgments

The authors thank BMCSCI (www.bmcscience.com) for the English language editing.

## Conflict of interest

The authors declare that the research was conducted in the absence of any commercial or financial relationships that could be construed as a potential conflict of interest.

## Publisher’s note

All claims expressed in this article are solely those of the authors and do not necessarily represent those of their affiliated organizations, or those of the publisher, the editors and the reviewers. Any product that may be evaluated in this article, or claim that may be made by its manufacturer, is not guaranteed or endorsed by the publisher.
